# Validation of an NSP-based (negative selection pattern) gene family identification strategy

**DOI:** 10.1186/1471-2105-9-S9-S2

**Published:** 2008-08-12

**Authors:** Ronald L Frank, Cyriac Kandoth, Fikret Ercal

**Affiliations:** 1 Biological Sciences Department, Missouri S&T, Rolla, MO 65409, USA; 2 Computer Science Department, Missouri S&T, Rolla, MO 65409, USA

## Abstract

**Background:**

Gene family identification from ESTs can be a valuable resource for analysis of genome evolution but presents unique challenges in organisms for which the entire genome is not yet sequenced. We have developed a novel gene family identification method based on negative selection patterns (NSP) between family members to screen EST-generated contigs. This strategy was tested on five known gene families in Arabidopsis to see if individual paralogs could be identified with accuracy from EST data alone when compared to the actual gene sequences in this fully sequenced genome.

**Results:**

The NSP method uniquely identified family members in all the gene families tested. Two members of the FtsH gene family, three members each of the PAL, RF1, and ribosomal L6 gene families, and four members of the CAD gene family were correctly identified. Additionally all ESTs from the representative contigs when checked against MapViewer data successfully identify the gene locus predicted.

**Conclusion:**

We demonstrate the effectiveness of the NSP strategy in identifying specific gene family members in Arabidopsis using only EST data and we describe how this strategy can be used to identify many gene families in agronomically important crop species where they are as yet undiscovered.

## Background

A significant proportion of genes that make up a genome are part of larger families of related genes resulting from duplications of individual genes [1], genomic segments [2], or even whole genomes ([3,4]. The accumulation of mutations in duplicates (paralogs) leads to either loss of function for one (death), altered function (subfunctionalization), or a new function (neofunctionalization). The study of the molecular processes by which functional innovation occurs interests not only evolutionary biologists, but protein engineers and medical and agricultural biologists. A clearer understanding of the extent to which gene families contribute to the selected traits in our most important crop species will help guide decisions regarding future improvements. Many studies are aimed at the diversity of function, expression, and regulation among gene family members in many species (reviewed in [5]). Others have spawned computational methods to analyze and predict the evolution of gene families in a phylogenetic context [6] or determine clinically relevant sites in a protein sequence where amino acid replacements are likely to have a significant effect on phenotype, including those that may cause genetic diseases [7].

Therefore, it is not surprising that research aimed at the identification of specific gene families and their constituent members has proliferated in the last few decades. Although experimental approaches using degenerate primers for PCR and oligofingerprinting [8] and cDNA library screening [9] generally produce the most reliable results, they can be time consuming and labor-intensive. Many strategies of gene family identification are computational approaches that take advantage of database mining and analysis tools to increase the capability and improve the efficiency of dealing with large amounts of sequenced data. Naturally, if a significant amount of a genome is sequenced computational methods can be somewhat more exhaustive in their search and identification [10-15]. However, complete genomic data is available for only a limited number of species. Expressed sequence tags (ESTs) on the other hand, are short, unedited, randomly selected single-pass sequences. They can be easily and inexpensively obtained directly from cDNA libraries. Although they were initially used for human gene discovery [16,17], exponential growth in the generation and accumulation of EST data for many diverse organisms has occurred in the last decade. The National Center for Biotechnology Information (NCBI) has a database for ESTs from over 1300 species totaling more than 48 million ESTs (as of 14 December 2007). Sixty-three species have more than 100,000 ESTs in the database making computational analyses more fruitful but complex. Because the number of ESTs in databases is increasing, computational techniques, including BLAST and its variants for comparative analysis and CAP3 [18] for sequence assembly, can be used to speed up gene or gene family identification processes and improve the feasibility of extracting meaningful information from a large and redundant database [19] when parameters are properly selected. These EST-based gene family identification strategies are valuable in species without fully sequenced genomes [20,21]. Caution must be exercised when assembling contigs from EST sequences because contigs not representative of real genes can result from chimera formation during cDNA cloning, errors in single-pass high-throughput sequencing of ESTs, or similarity between protein domains of unrelated sequences. Our group has developed a simple but novel method using evidence of negative selection pressure during divergence of the coding sequences to filter artifactual contigs from those potentially representing actual gene family members. Molecular evolution researchers studying divergence between well-characterized orthologs or paralogs often employ an estimation of the number of synonymous base substitutions per synonymous site versus the number of nonsynonymous base substitutions per nonsynonymous site [22,23]. A dS/dN ratio > 1 indicates purifying or negative selection (lower fitness) that tends to keep amino acid sequences the same if changes were deleterious. A ratio equal to 1 indicates changes that were neutral to fitness, while a dS/dN ratio < 1 would indicate adaptive or positive selection presumably because natural selection favored the amino acid changes. Differences between contigs that are artifactual should be proportionally distributed among synonymous and nonsynonymous sites, whereas differences between contigs that represent paralogs will often exhibit negative selection, dS/dN > 1.

We understand that negative selection may not be uniform over entire coding regions even assuming that purifying selection was at work in a given gene family. And not all gene families will exhibit negative selection between members. However, we believe that the number of gene families that can be detected by this approach is significant. Evidence has been found for a model whereby complementary deleterious mutations in regulatory elements between duplicate genes partitions the original function resulting in sub-functions [24]. It has also been discovered that the number of shared regulatory elements between duplicated genes in yeast decreases with evolutionary time [25]. The age of the duplicates was estimated by the accumulation of synonymous substitutions in the coding regions. Clearly, some forms of subfunctionalization can occur by changes in regulatory elements whereby some degree of negative selection has maintained protein function. Coding regions of paralogs that have subfunctionalized via changes to regulatory elements should exhibit a bias toward synonymous substitutions. In plants, a significantly greater proportion of genes belong to gene families than in animals or other major taxa [26]. Either gene duplication events have been more common in plants, or more duplicates have been retained during the evolutionary history of plants [27]. If this is the case, there should exist a significant number of gene families that can be identified by a bias toward synonymous substitutions when contigs are assembled from a significantly large database of ESTs. We have demonstrated previously that a simple strategy to detect negative selection patterns (NSP) among assembled ESTs provides a good screen for real versus artifactual contigs [28]. We have modified the filtering criterion to an empirically determined dS/dN threshold and decided to test the negative selection pattern (NSP) strategy on an EST database for which a large percentage of the ESTs have already been mapped to a fully-sequenced genome, *Arabidopsis thaliana*.

In this article we demonstrate the NSP strategy and report how well it was able to identify ESTs representing distinct family members in a genome where it is testable.

## Methods

### Gene family identification by NSP method

The five gene families chosen to validate the NSP strategy were, eukaryotic release factor 1 (*RF1*), ribosomal protein L6 (*L6*), cinnamyl alcohol dehydrogenase (*CAD*), phenylalanine ammonia-lyase (*PAL*), and an FtsH protease (*FtsH*). One member of the selected gene family was chosen as query for a tblastn search of the *Arabidopsis thaliana *dbEST. All hits with an E value < 1 × 10^-10 ^(maximum of 150 sequences) were selected and the resulting EST sequences were assembled using a contig assembly program (AssemblyLIGN, Oxford Molecular) with 100% match over a minimum 100 nucleotide overlap. The largest open reading frame greater than 100 codons was identified in each resulting non-singleton contig (MacVector, Accelrys). Open reading frames were translated and the resulting polypeptides aligned using ClustalX. The PAL2NAL program [29] produced a codon alignment of all contig open reading frames, and the SNAP program [30] at  was used to calculate dS/dN for all pairwise comparisons of contig open reading frames.

**Figure 1:**
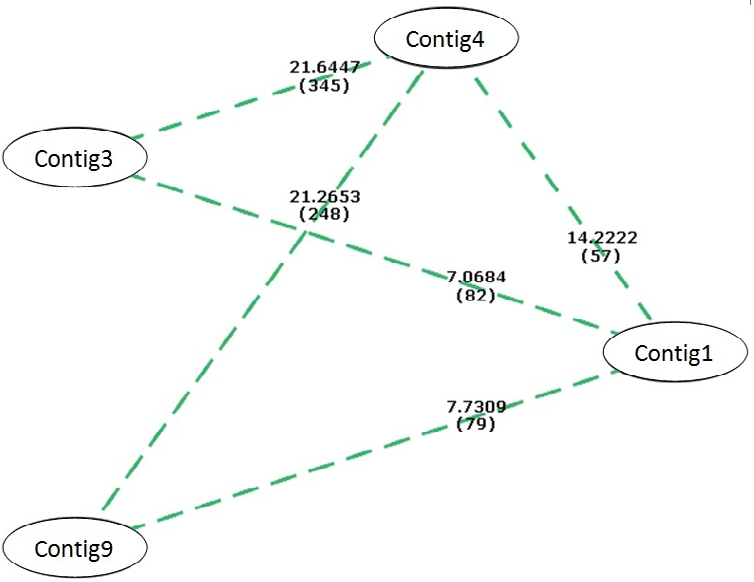
**Graph representing potential paralogs with dS/dN >= 2**. Edges are labeled with the dS/dN ratios, followed by the number of substitutions (Sd+Sn) seen An edge indicates dS/dN>=2.00; No edge indicates dS/dN<2.00 OR dS/dN=NA.

The empirically determined threshold for dS/dN was set to 2.00 and all pairs of contigs with a dS/dN ratio greater than this were classified as putative paralogs. A graph was constructed using vertices to represent contigs, and edges to represent whether pairs of contigs are putative paralogs. In such a graph, the largest fully connected sub-graph (the maximum clique) will be made up of vertices that represent markers (contigs) to the members of the same gene family as the query protein. This sub-graph was determined using a brute-force algorithm. A brute-force algorithm works by checking every possible sub-graph for connectedness. This operation is computationally expensive, and its time complexity increases exponentially, as the factorial of the number of vertices. Fortunately, the contigs that these vertices represent are usually quite few in number. Some contigs can also be excluded from the graph since they do not pass the dS/dN threshold to pair with any other contig. This can be observed in Figure 1 where only 5 pairwise comparisons of contigs obtained a dS/dN of more than 2.00.

Figure 1 shows the dS/dN ratios between contigs generated using the PAL1 gene as the protein query. Note that there are two maximum cliques in this graph. When there are more than one maximum cliques, we arbitrarily choose one of these cliques. The contigs represented by the vertices belonging to this clique are then identified as members of the same gene family. Any vertices that are not part of this clique are classified as either a possible marker to a distinct gene, or as a duplicate marker to an identified gene family member (in the maximum clique) which was different enough to be assembled into a different contig. In the case of Contig3 and Contig9 from Figure 1, it was found that these contigs were extremely similar to each other. They were later found to be duplicate markers to the PAL4 gene.

The representative contig for each putative gene family member identified was then compared to each of the actual gene family member sequences (NCBI) using bl2seq [31] to determine how closely contigs filtered through NSP represented the gene family. Either all or a subset of ESTs from each NSP-identified contig were checked on MapViewer (NCBI) to determine if ESTs from the same contig mapped to different gene family members or if ESTs from different contigs mapped to the same gene family member.

## Results

### Phenylalanine ammonia-lyase gene family

The tblastn search of using *AtPAL1 *protein as query resulted in ESTs and contigs reported previously [28]. Here we report the refinement of using dS/dN ratio rather than a tally of 1^st^, 2^nd^, and 3^rd ^position differences as well as the MapViewer results that validate the accuracy of gene family member identification. The dS/dN data for the assembled contigs are shown in Table 1 and the resulting maximum clique graph indicating putative paralog relationships is shown in Figure 1. The 2.0 dS/dN threshold was established empirically by dS/dN measurements among actual members of several Arabidopsis gene families. Pairwise comparison of contigs 1, 3, and 4 with the actual Arabidopsis gene sequences, reported previously [28] indicate that these three contigs represent AtPAL1, AtPAL4, and AtPAL2, respectively with greater than 96% similarity. The contigs selected by NSP as representative of real gene family members were further validated by checking to see if each EST comprising a single contig is assigned to a single gene family member on the Arabidopsis genome by NCBI MapViewer. Table 2 shows that all ESTs that comprise a single contig map to the same gene locus and confirms that contigs 1, 3, and 4 represent the PAL1, PAL4, and PAL2 genes of Arabidopsis, respectively.

**Table 1:** dS/dN calculations for phenylalanine ammonia-lyase (PAL) contigs

**Comparison**		**Sd^a^**	**Sn**	**S**	**N**	**ps**	**pn**	**ds**	**dn**	**ds/dn**	**ps/pn**
Contig1	Contig4	38.50	18.50	62.83	219.17	0.61	0.08	1.27	0.09	14.22	7.26
Contig1	Contig3	42.17	39.83	65.17	216.83	0.65	0.18	1.49	0.21	7.07	3.52
Contig1	Contig9	42.33	36.67	65.50	216.50	0.65	0.17	1.48	0.19	7.73	3.82
Contig1	Contig6	52.50	138.50	63.33	197.67	0.83	0.70	NA	0.00	NA	1.18
Contig1	Contig8	53.50	138.50	63.33	197.67	0.84	0.70	NA	0.00	NA	1.21
Contig1	Contig7	45.17	153.83	62.83	210.17	0.72	0.73	2.39	2.80	0.85	0.98
Contig4	Contig3	211.83	133.17	286.33	985.67	0.74	0.14	3.22	0.15	21.64	5.48
Contig4	Contig9	143.00	105.00	191.67	639.33	0.75	0.16	3.94	0.19	21.27	4.54
Contig4	Contig6	152.50	490.50	201.50	665.50	0.76	0.74	NA	0.00	NA	1.03
Contig4	Contig8	80.33	225.67	99.83	314.17	0.80	0.72	NA	0.00	NA	1.12
Contig4	Contig7	65.00	233.00	93.17	326.83	0.70	0.71	2.00	2.25	0.89	0.98
Contig3	Contig9	1.50	14.50	197.17	633.83	0.01	0.02	0.01	0.02	0.33	0.33
Contig3	Contig6	160.50	486.50	206.83	660.17	0.78	0.74	NA	0.00	NA	1.05
Contig3	Contig8	81.83	222.17	102.83	311.17	0.80	0.71	NA	0.00	NA	1.11
Contig3	Contig7	70.50	228.50	96.00	324.00	0.73	0.71	2.90	2.11	1.37	1.04
Contig9	Contig6	150.00	454.00	196.17	613.83	0.76	0.74	NA	0.00	NA	1.03
Contig9	Contig8	81.33	220.67	103.17	310.83	0.79	0.71	NA	0.00	NA	1.11
Contig9	Contig7	71.67	229.33	96.33	323.67	0.74	0.71	3.61	2.17	1.66	1.05
Contig6	Contig8	2.00	4.00	108.33	305.67	0.02	0.01	0.02	0.01	1.42	1.41
Contig6	Contig7	68.33	200.67	97.50	301.50	0.70	0.67	2.04	1.64	1.25	1.05
Contig8	Contig7	68.33	199.67	97.50	301.50	0.70	0.66	2.04	1.61	1.27	1.06

SNAP output results for all 21 pairwise comparisons of 7 contigs in which an ORF was identified. A ds/dn value greater than 2.00 was chosen as threshold to indicate contigs that potentially represent distinct gene family members.

a – See  for explanation of abbreviations and calculations.

**Table 2:** MapViewer locus for ESTs of NSP generated contigs

**Putative gene family**	**Gene group by NSP**	**Contig**	**EST accession**	**MapViewer locus**	**MapViewer Gene Name**
*CAD*	GeneB	contig3	CK121258	AT4G39330	AtCAD1
			CB074210	AT4G39330	AtCAD1
	GeneC	contig1	BP561562	ELI3-1	AtCAD4
			BP796450	ELI3-1	AtCAD4
			CD530744	ELI3-1	AtCAD4

*RF1*	GeneA	contig1	AV823314	ERF1-3	AteRF1-3
	GeneB	contig3	AV822373	ERF1-2	AteRF1-2
			BP803175	ERF1-2	AteRF1-2
			Z18188	ERF1-2	AteRF1-2
	GeneC	contig6	AV825957	ERF1-1	AteRF1-1
			BE845168	ERF1-1	AteRF1-1

*PAL*	GeneA	contig1	8720101	PAL1	AtPAL1
			8736225	PAL1	AtPAL1
	GeneB	contig3	8722848	AT3G10340	AtPAL4
			8723431	AT3G10340	AtPAL4
			8728745	AT3G10340	AtPAL4
			8730514	AT3G10340	AtPAL4
			9780248	AT3G10340	AtPAL4
			9788228	AT3G10340	AtPAL4
	GeneC	contig6	8690351	PAL2	AtPAL2
			8724245	PAL2	AtPAL2
			8725529	PAL2	AtPAL2
			19869024	PAL2	AtPAL2
			19869200	PAL2	AtPAL2
			37426635	PAL2	AtPAL2
	GeneC	contig8	9786707	PAL2	AtPAL2
			37426640	PAL2	AtPAL2
	GeneC	contig4	8719100	PAL2	AtPAL2
			14580232	PAL2	AtPAL2
			19855615	PAL2	AtPAL2
			49165014	PAL2	AtPAL2
			59667557	PAL2	AtPAL2

*L6*	GeneA	contig1	5761694	AT1G18540	AtL6A
			8724065	AT1G18540	AtL6A
			19802678	AT1G18540	AtL6A
	GeneB	contig4	19868834	AT1G74060	AtL6B
			23303389	AT1G74060	AtL6B
	GeneC	Contig6	8714872	AT1G74050	AtL6C

*FtsH*	GeneA	contig1	AV518555	VAR2	AtFtsH2
			AV558102	VAR2	AtFtsH2
			AV800962	VAR2	AtFtsH2
			BP785237	VAR2	AtFtsH2
	GeneB	contig6	BP626558	FTSH8	AtFtsH8

Individual ESTs of representative contigs for putative gene family members of the 5 Arabidopsis families tested were located to a specific locus by NCBI MapViewer.

For the following four additional NSP-identified gene families only the validating data is shown, not the dS/dN data or maximum clique graphs.

### Ribosomal protein L6 gene family

*AtRPL6A *protein was used as query for the tblastn search of *A. thaliana *dbEST yielding 150 EST sequences that assembled into eight contigs ranging from 449 to 953 bases and 2 to 36 ESTs each. Following ORF identification the 28 pairwise codon alignments and subsequent dS/dN values were analyzed to sort contigs into putative gene family members. From that analysis contig1, contig3 and contig8 were assigned to putative geneA, contig2, contig4, and contig5 to geneB, and contig6 to geneC. Table 3 shows that each of these contig groups identified, by greater than 98% similarity, a different member of the Arabidopsis ribosomal protein *L6 *gene family when aligned to the actual gene sequences.

**Table 3:** Percent similarity for NSP generated contigs aligned with actual ribosomal protein L6 genes

	**GeneA**			**GeneB**			**GeneC**
	**contig1**	**contig3**	**contig8**	**contig2**	**contig4**	**contig5**	**contig6**

*AtL6A*	100	98	98	82	83	82	84
*AtL6B*	83	83	82	99	99	99	93
*AtL6C*	84	84	83	93	93	93	99

Representative contigs for 3 putative gene family members, GeneA, GeneB, and GeneC, identified by the NSP method were aligned with actual Arabidopsis gene family members and percent similarity determined.

In Table 2 for ribosomal protein L6 it can be seen that all ESTs from the same contig as well as all ESTs from the same gene grouping are assigned to the same gene locus. Also, in no instances did ESTs belonging to different gene groupings by NSP ever map to the same gene locus.

### Cinnamyl alcohol dehydrogenase gene family

The tblastn search of using *AtCAD5 *protein as query resulted in 150 EST sequences. The ESTs assembled into eight contigs ranging from 592 to 1248 bases and 2 to 21 ESTs each. Following ORF identification the 28 pairwise codon alignments and subsequent dS/dN values were analyzed to sort contigs into putative gene family members as described above (data not shown). The eight contigs assorted into four groups based on their negative selection pattern with each other contig. These four groups were arbitrarily designated GeneA represented by contig4, contig6, and contig8, GeneB, represented by contig3, contig5, and possibly contig7, GeneC represented by contig1, and GeneD represented by contig2.

The results of the comparison of representative contigs to the actual gene sequences for the CAD gene family of Arabidopsis are shown in Table 4. Each contig group identified, by greater than 99% similarity, a different member of the CAD gene family. MapViewer analysis for the CAD gene family (Table 2) shows that all ESTs from the same contig are assigned to the same gene locus, and no ESTs belonging to different contigs map to the same gene locus. Contigs validated by alignment to actual genes but not shown in Table 2 are comprised of ESTs that have not yet been mapped to specific loci by MapViewer.

**Table 4:** Percent similarity for NSP generated contigs aligned with actual CAD genes

	**GeneA**	**GeneB**	**GeneC**	**GeneD**
	**contig8**	**contig3**	**contig1**	**contig2**

*AtCAD-1*	NSS^a^	99	NSS	NSS
*AtCAD-2*	99	NSS	NSS	NSS
*AtCAD-3*	NSS	NSS	78	82
*AtCAD-4*	NSS	76	100	87
*AtCAD-5*	NSS	72	84	100
*AtCAD-6*	79	NSS	NSS	NSS
*AtCAD-7*	NSS	78	72	NSS
*AtCAD-8*	NSS	NSS	NSS	NSS
*AtCAD-9*	NSS	NSS	NSS	NSS

Representative contigs for 4 putative gene family members, GeneA, GeneB, GeneC, and GeneD identified by the NSP method were aligned with actual Arabidopsis gene family members and percent similarity determined.

a – No significant similarity as returned by bl2seq program.

### Release Factor 1 gene family

*AtRF1-3 *protein was used as query for the tblastn search of *A. thaliana *dbEST yielding 109 EST sequences that assembled into six contigs ranging from 591 to 930 bases and three to 19 ESTs each. Following ORF identification the 15 pairwise codon alignments by the NSP program resulted in three contigs exhibiting NSP. These were arbitrarily assigned as contig1 representing geneA, contig3 representing geneB, and contig6 representing geneC. Each of these contigs identified, by greater than 97% similarity, a different member of the Arabidopsis RF1 gene family when aligned to the actual gene sequences (Table 5). MapViewer results again show that ESTs comprising NSP-selected contigs are unambiguous in the gene locus to which they have been assigned (Table 2).

**Table 5:** Percent similarity for NSP generated contigs aligned with actual release factor genes

	**GeneA**	**GeneB**	**GeneC**
	**contig1**	**contig3**	**contig6**

*AtRF1-1*	82	83	99
*AtRF1-2*	88	97	83
*AtRF1-3*	99	85	82

Representative contigs for 3 putative gene family members, GeneA, GeneB, and GeneC, identified by the NSP method were aligned with actual Arabidopsis gene family members and percent similarity determined.

### FtsH protease gene family

The TBLASTN search of using *AtFtsH8 *protein as query resulted in 150 EST sequences. The ESTs assembled into six contigs ranging from 526 to 1217 bases and 2 to 33 ESTs each. Following ORF identification the 15 pairwise alignments by the NSP program resulted in two contig groups exhibiting NSP. Contig1 and contig5 represent geneA, and contig3 and contig6 represent geneB. Each of these contig groups identified, by greater than 97% similarity, a different member of the Arabidopsis *FtsH *gene family when aligned to the actual gene sequences, as shown in Table 6. MapViewer results again show that ESTs comprising NSP-selected contigs are unambiguous in the gene locus to which they have been assigned (Table 2).

**Table 6:** Percent similarity for NSP generated contigs aligned with actual FtsH genes

	**GeneA**		**GeneB**	
	**contig1**	**contig5**	**contig3**	**contig6**

*AtFtsH1*	NSS	71	73	NSS
*AtFtsH2*	100	97	86	83
*AtFtsH3*	NSS	78	70	NSS
*AtFtsH4*	NSS	79	78	NSS
*AtFtsH5*	NSS	73	73	NSS
*AtFtsH6*	72	73	69	77
*AtFtsH7*	NSS	68	73	NSS
*AtFtsH8*	88	85	99	100
*AtFtsH9*	NSS	68	NSS	NSS
*AtFtsH10*	NSS	75	74	NSS
*AtFtsH11*	NSS	77	77	NSS
*AtFtsH12*	NSS	NSS	NSS	NSS

Representative contigs for 2 putative gene family members, GeneA and GeneB, identified by the NSP method were aligned with actual Arabidopsis gene family members and percent similarity determined.

## Discussion

It has been observed for some time that contig assembly from EST sequences can produce artifactual sequences resulting from relatively high error in EST sequences, chimeras generated in cDNA cloning, and regions of highly conserved domains interspersed in related genes. Therefore, it is necessary that strategies involving the generation of contigs from ESTs employ some criterion for either eliminating unauthentic coding regions or selecting for authentic ones. We have found that contigs representing gene families where the paralogous coding regions have been constrained by negative (purifying) selection pressure can be identified by screening for amino acid substitution patterns indicative of such (NSP, Negative Selection Patterns). However, if differences between contigs are artifacts no pattern among codon positions should be exhibited. If no negative selection pattern is detected we do not conclude that the contigs necessarily represent the same gene. Our goal is only to identify contigs that represent different genes of the same family. We do not expect that all members of a particular family will be detectable by this method. Other members may be identified with iterative searches using previously identified contigs.

To illustrate that this method can identify members of a gene family with some accuracy using only EST data we tested it on five well-characterized gene families in Arabidopsis. Each case resulted in successful identification of one to three additional gene family members distinct from the member used as initial query. Of the eight initial contigs generated from EST hits when *AtCAD5 *was used as query the NSP strategy identified those representing *AtCAD1*, *AtCAD2*, and *AtCAD4*, in addition to one representing *AtCAD5 *(Table 4). Moreover, each of these contigs exhibited less than 87% similarity to other actual members of the gene family. No contigs generated at the parameters specified in the assembly program represented *AtCAD3*, 6, 7, 8 or 9. This could be the result of relative expression levels of those genes, limits on the necessary similarity between gene family members, or limitations on the method which are discussed elsewhere [28]. Similarly, when ribosomal protein *L6A *was used as query the NSP strategy identified contigs accurately representing all three genes of the family, *L6A*, *L6B*, and *L6C *(Table 3). Furthermore, all three members of the *RF1 *gene family were accurately represented by NSP-screened contigs (Table 5), as were *AtFtsH2 *and *AtFtsH8 *of that 12-member gene family (Table 6). We previously reported the accurate identification of *AtPAL1*, 2, and 4 of phenylalanine ammonia-lyase gene family and show here further validation that the contigs identified the appropriate gene family members.

In addition, we were able to show that all the ESTs of a single contig defined the same actual gene family member according to MapViewer (Table 2), i.e., all ESTs of a single contig mapped to the same locus, and perhaps more importantly, no ESTs from different contigs of the same gene family ever mapped to the same locus. This would suggest that although the initial assembly of related ESTs may indeed generate non-valid contigs, screening by NSP allows one to determine which contigs represent real gene loci.

A limitation to the NSP strategy is the fact that only paralogs that exhibit purifying selection can be identified and that selection pattern must be evident in the portion of the coding region reconstructed by contig assembly, roughly the 3' two-thirds of the protein by our experience. For this reason the NSP strategy in it current phase will only identify a subset of gene families. However, when we consider that estimates of the number of gene families in a plant species may be 10–12,000 [32], that subset may comprise a significant portion in which NSP can detect two to three additional family members. Our goal is to broaden the NSP approach to identify as many gene families as possible without sacrificing the accuracy reported here. We have already automated the four basic steps, 1) BLAST collection of related ESTs, 2) contig assembly, 3) ORF identification, and 4) NSP screening of contigs, such that the input is a query protein of a potential gene family member and the output is contigs representing at least two gene family members. Since the query can be an orthologous sequence, we are currently working on identifying, in *Glycine max*, every gene family for which at least one member has been identified in another plant species. The specific objectives for accomplishing this are to:

1) use all known *Glycine max *mRNAs as queries to identify other family members, if any.

2) use mRNAs from related species as queries to identify gene families not identified above.

3) use Arabidopsis gene families as queries (currently about 1000 gene families in TAIR).

4) use other Arabidopsis genes, not currently associated with a family as queries to identify potential genes existing as a family in soybean but not so in Arabidopsis.

5) use all *Glycine max *ESTs not included in contigs from above searches in clustering experiments to potentially identify novel gene families.

Objectives 1–4 above are identical in protocol. They differ only in the species of origin for the protein query. There are currently about 1350 known *Glycine max *gene sequences in the NCBI database, mostly mRNA sequences but some genomic. Some of these already represent multiple members of the same gene family (e.g. glycinin and conglycinin seed storage proteins, uricase, ascorbate peroxidase, lipoxygenase, rubicase small subunit, phosphoenolpyruvate carboxylase, etc) [33]. Objective 1 will use all known genes of soybean as queries to identify other members of the gene family. Objective 2 involves genes from species more closely related to soybean than Arabidopsis. These include other eurosids I and particularly other legumes that have significant sequence data available such as *Pisum sativum, Phaseolus vulgaris*, and *Medicago truncatula*. Objective 3 will involve queries chosen from Arabidopsis genes that are known to exist as part of a gene family. Currently, The Arabidopsis Information Resource (TAIR) has genomic, coding region, and amino acid sequence data for 996 gene families comprised of 8,331 genes. Objective 4 will use as initial queries all remaining Arabidopsis genes not already identified in soybean and not associated with a gene family in TAIR. It is possible that of the remaining 16,000 genes of Arabidopsis there could be some that are associated with a family in *Glycine max*. Objective 5 does not start with a query sequence but rather a set of ESTs clustered by similarity to each other. Several clustering algorithms could be used for this, UniGene (at NCBI), PACE [34], or one developed in our laboratory several years ago. The majority of UniGene clusters are annotated with "strongly similar to," moderately similar to," or "weakly similar to" gene or protein functions of other organisms. Others are labeled simply as "Transcribed locus" to indicate that they represent RNA sequences that do not show similarity to any currently known gene or protein (Build #31 has 6812 such clusters). We have run a few of these clusters through the NSP strategy and found that some will generate contigs that indicate the cluster may represent ESTs from distinct members of a gene family. More work in this direction will allow us to expand the strategy to include identification of yet undiscovered gene families.

## Conclusion

Although the NSP strategy is not a global gene family identification protocol, our tests on the Arabidopsis EST dataset indicate that it performs well in distinguishing contigs that represent real genes from contigs that are artifacts. Every EST tested, from contigs that NSP predicted to be distinct gene family members, mapped to the appropriate gene in Arabidopsis. Further expansion of the strategy to clustered ESTs eliminating the need for individual query sequences and further automation of the steps will allow the identification of a significant proportion of gene families with reliable accuracy.

## Competing interests

The authors declare that they have no competing interests.

## Authors' contributions

RLF participated in the conception and design of the study, carried out the gene family identification via NSP including BLAST, contig assembly, ORF identification, alignment and dS/dN analysis, and drafted the manuscript. CK developed scripts to construct graphical output of dS/dN results and performed all genome locus identification studies using MapViewer. FE participated in the conception, design, and development of the computational aspects of data generation. All authors read and approved the final manuscript.
